# Morphological Similarities of Chronic Ankle Instability and Cavus Foot Type Using Statistical Shape Modeling

**DOI:** 10.1002/jor.70128

**Published:** 2025-12-31

**Authors:** E. Renae Lapins, Alayna Fendler, Scott LaTulip, Devon C. Nixon, Amy L. Lenz

**Affiliations:** ^1^ Department of Biomedical Engineering University of Utah Salt Lake City Utah USA; ^2^ Department of Mechanical Engineering University of Utah Salt Lake City Utah USA; ^3^ School of Medicine University of Utah Salt Lake City Utah USA; ^4^ Orthopaedic Specialists of North Carolina Raleigh North Carolina USA; ^5^ Department of Orthopaedics University of Utah Salt Lake City Utah USA

**Keywords:** chronic ankle instability, foot types, pes cavus, statistical shape modeling, weightbearing computed tomography

## Abstract

Chronic ankle instability (CAI) can develop in up to 40% of patients after a first‐time ankle sprain. Advanced imaging and statistical shape modeling (SSM) provide the opportunity to evaluate if subtle differences in foot and ankle morphology, such as hindfoot alignment or cavus features, may contribute to structural predispositions for recurrent instability. In this study, a 14‐bone SSM was created from weight‐bearing CT data (*n* = 80) from patients with CAI (*n* = 23), cavus foot type (*n* = 29), and rectus (*n* = 28) foot type. Scans for the CAI group were from pre‐operative imaging of patients undergoing surgical stabilization. Meary's angle (MA), hindfoot ankle alignment (HAA), and calcaneal inclination were measured for each scan using in‐house code. Principal component analysis revealed that arch height (Mode 1, 36.9% of the variance) statistically distinguished the CAI and cavus groups from the rectus group. Minimal differences were observed between the CAI and cavus groups, with only Mode 5 (4.47% variance, $\eta^{2}$ = 0.09) separating the groups and Hotelling's T^2^ confirming minimal variation (~2% of particles). Radiographic measurements supported these findings with higher MA in CAI (12°) and cavus (17°) versus rectus (0.2°), and varus HAA in CAI (6.0°) and cavus (5.9°) compared to rectus (9.8°). Individuals with CAI demonstrated cavus‐like morphology, indicating that bony alignment may be a structural contributor to recurrent instability. Clinically, these findings enhance our understanding that foot alignment plays a role in CAI. Surgical correction, when clinically appropriate, may need to address both osseous deformities and soft tissue laxity to improve stability. Level III Orthopedic Research Society.

## 1 Introduction

Chronic Ankle Instability (CAI) can impact up to 40% of individuals following a first‐time lateral ankle sprain (LAS) [1]. Defined by recurrent sprains and persistent functional limitations lasting more than a year after the initial injury, CAI can impair mobility and diminish quality of life [2]. Although conservative management is effective for treating many LAS patients, some individuals continue to experience instability and may ultimately require surgical intervention where the lateral soft tissue structures are re‐tensioned. Repairs such as these (i.e., Brostrom‐Gould) are largely successful but can be less effective in individuals with generalized ligamentous laxity, longstanding instability, or high functional demands [3, 4, 5, 6]. Bony contributors to the development of ankle instability, particularly cavus foot morphology and hindfoot varus alignment, have been identified in CAI patients [7, 8, 9]. Fuller proposed that a rigid, supinated hindfoot with a laterally positioned subtalar axis increases the supination moment, thereby predisposing the ankle to recurrent inversion injury [10, 11, 12]. The cavus foot type with a varus hindfoot aligns with this mechanism, providing a possible structural explanation for recurrent sprains and instability [11, 13, 14]. These findings suggest that osseous alignment, along with ligamentous insufficiency, may predispose some patients towards CAI.

Traditionally, assessment of the foot and ankle morphology in CAI has relied on two‐dimensional (2D) radiographs and planar measurements such as Meary's angle (MA) or hindfoot alignment angle (HAA) [14, 15]. Although these methods provide valuable data, they offer a limited perspective on the complex three‐dimensional (3D) morphology of the foot and ankle. Subtle deviations in arch heights, hindfoot varus/valgus, or forefoot orientation may not be apparent on 2D radiographs yet could meaningfully influence joint loading and instability risk. Weight‐bearing computed tomography (WBCT) provides static, high‐resolution three‐dimensional (3D) imaging of the foot and ankle under physiological load and captures bony characteristics that may not be detectable or obvious on conventional radiographs [16, 17, 18].

Statistical shape modeling (SSM) further enhances the use of WBCT data by allowing a quantitative analysis of bony morphology across populations. SSM has been successfully applied in the foot and ankle to assess a variety of different pathologies and identify subtle morphological variations [19, 20, 21, 22]. By combining WBCT with SSM, it is possible to evaluate the structural characteristics of CAI patients and evaluate the subtle shape differences that may not be captured by traditional radiographic measurements.

Cavus foot type, characterized by a high medial longitudinal arch and hindfoot varus, is associated with lateral ligament overload and instability [23, 24, 25]. In contrast, the rectus foot type represents a neutral alignment, providing a structural baseline for comparison. Identifying if patients with CAI demonstrate morphological similarities to specific foot types could provide insight into structural predispositions that contribute to recurrent instability and may inform individualized approaches to surgical management.

The purpose of this study was to compare the morphology between individuals with CAI, cavus foot, and rectus foot type using WBCT and SSM. The CAI cohort included patients undergoing surgical correction for instability who had pre‐operative WBCT scans performed. Cavus and rectus patients were selected from a larger database of controls. Radiographic measurements were also calculated from 3D segmentations to complement shape analysis and quantify key alignment parameters, including MA, HAA, and calcaneal inclination (CI). We hypothesized that individuals with CAI would demonstrate structural characteristics consistent with cavus morphology, supporting the concept that bony alignment may contribute to the development of CAI.

## 2 Methods

### 2.1 Patient Data and Demographics

All patient WBCT data (0.37 mm^3^ voxels) from 80 patients (28 rectus, 23 CAI, and 29 cavus feet) were acquired retrospectively with IRB approval from foot and ankle orthopedic examinations performed between 2016 and 2022 (Table 1). Inclusion criteria included CAI patient scans from patients undergoing surgical intervention for instability, such as a Brostrom or a combined Brostrom and deltoid repair, who also had preoperative WBCT scans available. Exclusion criteria consisted of: previous surgical repair or interventions, ankle or midfoot fusions, osteoarthritis, and fractures. Rectus and cavus individuals consisted of retrospectively identified contralateral limbs from acute unilateral foot injuries, with foot type classified through musculoskeletal assessment of weight‐bearing radiographs performed by a radiologic technologist, followed by computationally derived MA values that validated these classifications. MA measurements were obtained using an in‐house MATLAB toolkit that mathematically derives common radiographic measurements from 3D bony segmentations. Rectus MA range was −4.5° ≤ MA ≤ 4.5°, and cavus MA included any MA values greater than 4.5° [25, 26]. A cutoff value of 4.5° was used to account for rounding.

**Table 1:** Patient demographic information.

	Rectus	Cavus	CAI	All data
Ankles (#)	28	29	23	80
Mean age ± SD (years), range	40 ± 18 (19–74)	40 ± 17 (17–69)	37 ± 13 (14–64)	39 ± 16 (14–74)
Sex (%), male/female	39/61	66/34	52/48	51/49
Laterality (%), left/right	61/39	45/55	46/54	46/54

### 2.2 Data Processing

WBCT scans were semi‐automatically segmented using commercial software (DISIOR Bonelogic 2.0; Paragon28, Helsinki, Finland), producing 3D segmentations of the calcaneus, cuboid, intermediate/lateral/medial cuneiforms, metatarsals 1 through 5, navicular, talus, and tibia. The initial segmentations were then manually reviewed, refined, smoothed, and decimated using Mimics 24.0 and 3‐Matic 16.0 (Materialise, Leuven, Belgium) to ensure consistent mesh quality across all reconstructed bones. To standardize anatomical orientation, all left‐foot data were mirrored to match right‐sided anatomy. Iterative closest point alignment was applied in MATLAB 2024a (MathWorks Inc., Natick, MA, USA) to align the 14 segmented bones of each patient with those of another. The aligned and smoothed 3D bone meshes were used as inputs for SSM, following protocols established in prior studies [20]. These bony segmentations also served as inputs for our in‐house radiographic measurement toolkit, which computationally calculates MA, HAA, and CI from the 3D meshes [27]. Sagittal MA assesses the general arch height of the individuals and describes the general foot type. HAA explains the orientation of the hindfoot in relation to the tibia and is commonly used in clinical assessment of instability. CI describes the pitch of the calcaneus bone in the sagittal plane and is used to assess the height of the medial arch.

### 2.3 SSM

A 14‐bone SSM was created from the pre‐processed meshes in ShapeWorks 6.6.0 (SCI Institute, Salt Lake City, UT, USA), an open‐source SSM software [28]. This software employs a particle‐based shape modeling technique where correspondence points are represented by dynamic sets of interacting particles. Through an energy‐driven optimization process, these particles adjust their position to preserve anatomical correspondence across subjects and to precisely capture the bone surface geometry. The number of particles assigned to each bone varied, ensuring consistent surface coverage and accurate representation of each bone's geometry. During optimization, a generalized Procrustes analysis was applied to scale each patient's bony anatomy, ensuring that inherent foot size differences did not interfere with the variation analysis. Once good particle correspondence was reached, statistical analyses were conducted.

### 2.4 Statistical Analyses

Principal component analysis (PCA) evaluated the modes of variation from the SSM model correspondence particle locations. To determine which modes were statistically meaningful to retain, parallel analysis was conducted [29]. The Kolmogorov–Smirnov test (α = 0.05) was applied to assess the normality of PCA score distributions across modes [30]. Since all PCA modes followed a normal distribution, a one‐way ANOVA was performed on the retained modes to identify statistical differences among patient groups, followed by a Tukey's post hoc test (α = 0.05). Effect size ($\eta^{2}$) was calculated for each retained mode using ANOVA results and interpreted according to Cohen's benchmarks: 0.01 small, 0.06 medium, and 0.14 for large effects [31, 32]. To evaluate differences in radiographic measurements calculated (MA, HAA, and CI), Welch's ANOVA with Games‐Howell post hoc (α = 0.05) and Hedge's *g* effect size were implemented to assess differences, accounting for unequal variances across groups [33, 34, 35]. Conventional thresholds were used to assess effect size results: small effects (*g* > 0.20), medium effects (*g* > 0.50), and large effects (*g* > 0.80). To identify localized regions with significant differences in both overall morphology and alignment, a Hotelling's T^2^ test was used with a false discovery rate *p*‐value correction. *p*‐values in this test are calculated between each correspondence particle of the group mean shapes in the SSM (α = 0.05) and separated into shape and/or alignment differences depending on local or world particle location differences.

## 3 Results

### 3.1 SSM and PCA

**Figure 1:**
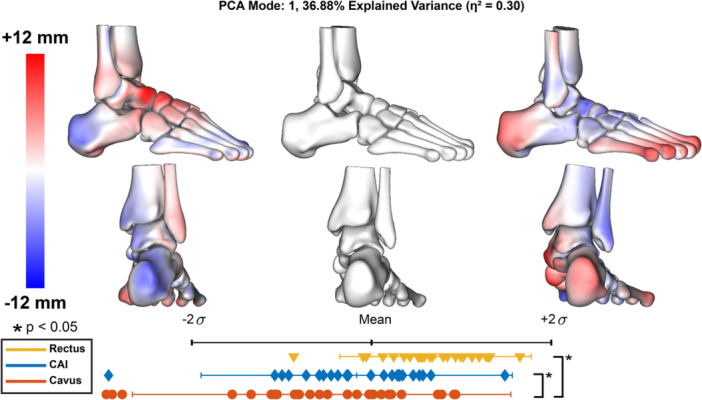
PCA mode 1 results. Surface distances are displayed within the two standard deviations, indicating the variation from the mean shape (center; gray), measured in millimeters (mm). Red indicates shape differences away from the bone, representing an increase in feature size, while blue indicates shape differences toward the bone, representing a decrease. Each marker represents a patient in each group and their corresponding component score along the two standard deviation distribution range. Significant differences between groups were determined by one‐way ANOVA and Tukey post hoc analysis (*α* = 0.05) and are indicated by stars. Effect size ($\eta^{2}$) was calculated for each retained mode using ANOVA results.

**Figure 2:**
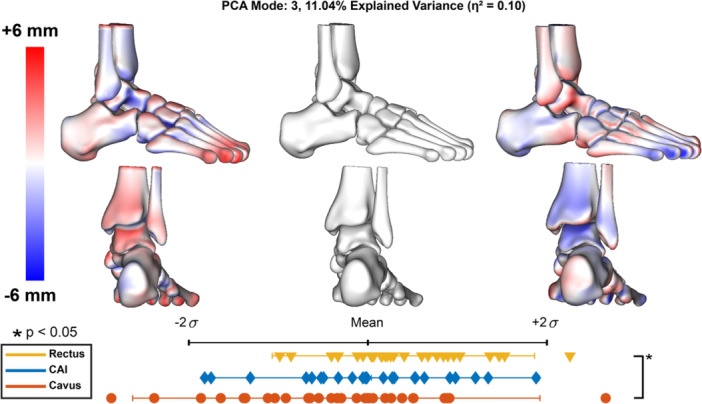
PCA mode 3 results. Surface distances are displayed within the two standard deviations, indicating the variation from the mean shape (center; gray), measured in millimeters (mm). Red indicates shape differences away from the bone, representing an increase in feature size, while blue indicates shape differences toward the bone, representing a decrease. Each marker represents a patient in each group and their corresponding component score along the two standard deviation distribution range. Significant differences between groups were determined by one‐way ANOVA and Tukey post hoc analysis (*α* = 0.05) and are indicated by stars. Effect size ($\eta^{2}$) was calculated for each retained mode using ANOVA results.

**Figure 3:**
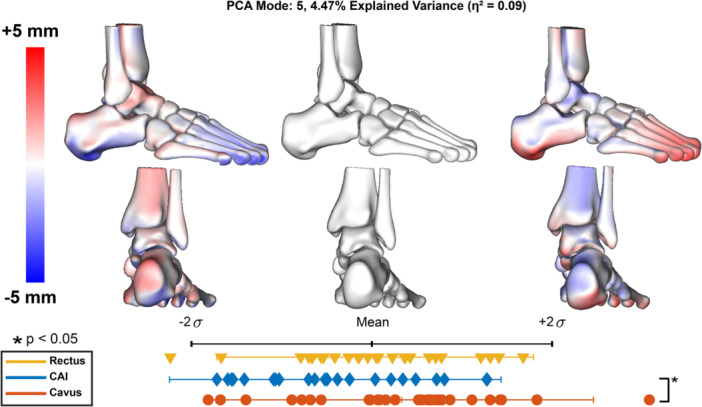
PCA mode 5 results. Surface distances are displayed within the two standard deviations, indicating the variation from the mean shape (center; gray), measured in millimeters (mm). Red indicates shape differences away from the bone, representing an increase in feature size, while blue indicates shape differences toward the bone, representing a decrease. Each marker represents a patient in each group and their corresponding component score along the two standard deviation distribution range. Significant differences between groups were determined by one‐way ANOVA and Tukey post hoc analysis (*α* = 0.05) and are indicated by stars. Effect size ($\eta^{2}$) was calculated for each retained mode using ANOVA results.

Parallel analysis identified nine modes to retain, which captured 87% of the overall variance. Of these modes, only three modes included statistical differences between group distributions (Figures 1, 2, 3).

PCA mode 1 accounted for 37% of the overall variance and represented foot type, ranging from cavus to rectus alignment (Figure 1). In this mode, a raised medial longitudinal arch and varus hindfoot alignment are seen at −2 standard deviations (σ) below the mean shape. Oppositely, a neutral arch and more valgus hindfoot alignment is evident at +2σ above the mean shape. The rectus group distribution aligned closer to +2σ, and the cavus and CAI groups clustered closer to the mean shape and −2σ. Significant morphological differences were observed between the rectus and cavus groups, as well as between the rectus and CAI groups. This mode has a large effect size ($\eta^{2}$) of 0.30.

PCA Mode 3 represented 11% of the variance and had a large effect size of 0.10 (Figure 2). This mode only had a statistical difference between the rectus and cavus groups. The cavus group aligned more with +2σ where we see a higher medial arch with increased CI and plantarflexion of the first ray. At +2σ, the foot shape has a collinear talus and first‐metatarsal and a neutral arch.

PCA mode 5 accounted for 4% of the overall variance and had a medium effect size of 0.09 (Figure 3). In this mode, we see differences mainly in the calcaneus and metatarsals, where the calcaneal pitch is raised at +2σ and lower at −2σ. The tibia is also shifted anteriorly in the +2σ shape. There were statistical differences only between the CAI and Cavus group indicated by a star. Effect size ($\eta^{2}$) was calculated for each retained mode using ANOVA results.

### 3.2 Hotelling's T^2^ Analysis

**Figure 4:**
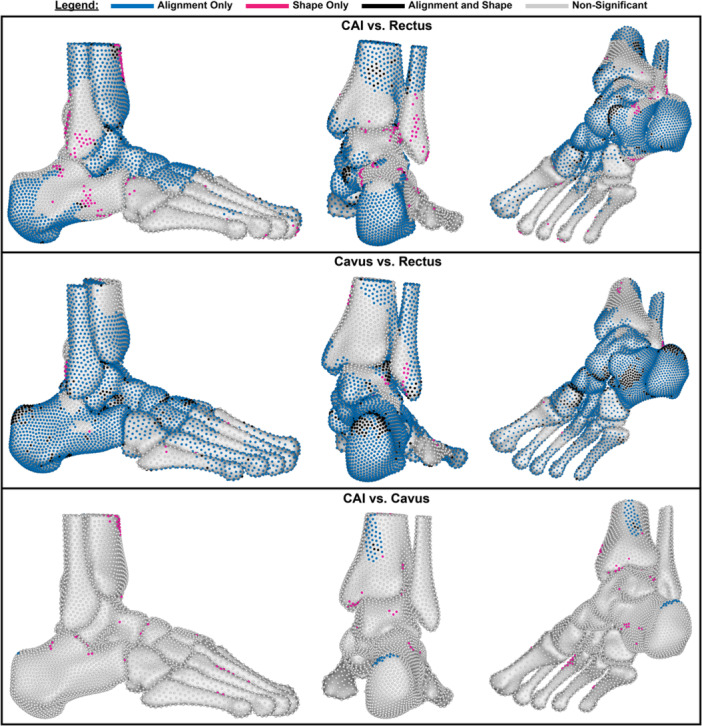
Hotelling's T^2^ analysis results comparing the different group mean shapes. Top row: CAI versus Rectus (49% alignment only, 6% shape only, 4% alignment and shape). Middle row: Cavus versus Rectus (68% alignment only, 9% shape only, 9% alignment and shape). Bottom row: CAI versus Cavus (1% alignment only, 2% shape only, 0.04% alignment and shape).

When comparing the group means of CAI and cavus with rectus foot type, we see mostly alignment‐only significant differences (Figure 4). Shape differences are minimal in all group mean comparisons. Cavus versus rectus comparison yields more significantly different particles in both shape and alignment compared to the CAI versus rectus comparison. The comparison of the CAI group to the cavus group yielded minimal differences.

### 3.3 Radiographic Measurement Analyses

**Figure 5:**
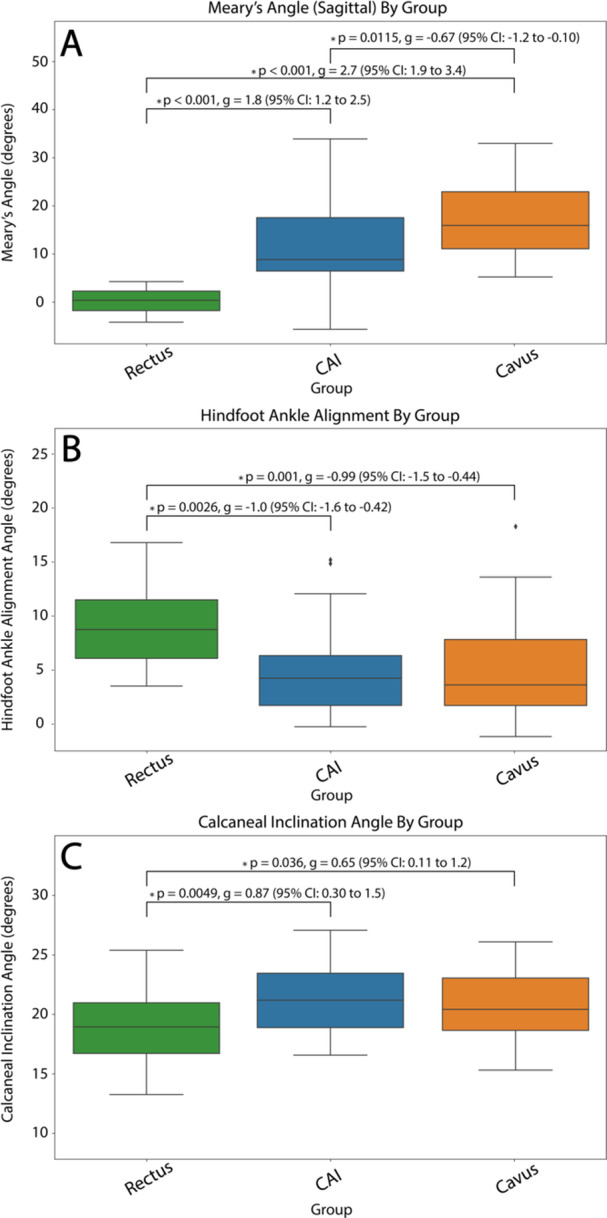
Radiographic measurement comparisons between rectus, chronic ankle instability (CAI), and cavus patient groups. (A) Meary's angle results. (B) Hindfoot ankle alignment angle results. (C) Calcaneal inclination angle results.

Mean radiographic measurements and ranges for each group are shown in Table 2. There were significant differences between all three groups when comparing MAs to each other (Figure 5A). A large effect size was observed when comparing the rectus group to both the CAI and cavus groups, whereas the comparison between the CAI and cavus groups demonstrated only a medium effect. HAA and CI values were significantly different between the rectus group and both the CAI and Cavus groups (Figure 5B,C). No statistical differences were found between the CAI and cavus group HAA and CI measurements. The only significant difference between the CAI and cavus group was observed in the MA assessment.

**Table 2:** Radiographic measurements for each group.

	Rectus	Cavus	CAI	All data
Mean Meary's angle ± SD (degrees), range	0.2 ± 2.6	17 ± 8.6	12 ± 8.6	9.7 ± 10
	Min: −4.2	Min: 5.2	Min: −5.7	Min: −5.7
	Max: 4.3	Max: 33	Max: 34	Max: 34
Mean Hindfoot ankle alignment ± SD (degrees), range	9.8 ± 3.4	5.9 ± 4.1	6 ± 3.9	7.3 ± 4.2
	Min: 4.6	Min: 0.13	Min: 0.99	Min: 0.13
	Max: 17	Max: 19	Max: 16	Max: 19
Mean Calcaneal inclination angle ± SD (degrees), range	19 ± 3.6	21 ± 3.1	22 ± 3.1	20 ± 3.4
	Min: 9.0	Min: 15	Min: 17	Min: 9
	Max: 25	Max: 26	Max: 27	Max: 27

## 4 Discussion

Individuals with CAI who required surgical correction exhibited foot morphology that closely resembled the cavus foot type, confirming our hypothesis that bony alignment may play a role in the development of recurrent instability. Recognizing cavus‐like structural features can help identify patients at a higher risk of developing CAI. In selected patients, surgical strategies should consider both osseous realignment and soft tissue reconstruction.

Prior literature has linked cavus foot morphology and hindfoot varus to lateral ankle instability [7, 8, 9]. Specifically, hindfoot varus and increased arch height have been associated with altered peroneal mechanics and greater lateral column loading [8, 24]. Quantitative imaging studies using WBCT further support this relationship, demonstrating a tendency toward varus hindfoot alignment in patients with CAI compared to rectus [8]. SSM results from this study provide quantitative evidence that supports these relationships from the literature. PCA mode 1 demonstrated differences in arch height and clearly distinguished both the CAI and cavus groups from rectus, suggesting that an elevated medial arch is a dominant feature of CAI morphology. Only PCA mode 5, which accounted for 4.47% variance, statistically differentiated the CAI from the cavus group, and Hotelling's T^2^ analysis identified negligible differences (~2% of significantly different correspondence particles) between the two groups.

Radiographic measurements from this study reinforce the SSM results. MA values identified an elevated arch in both the CAI (12°) and cavus (17°) groups, and HAA values shifted toward varus in CAI (6.0°) and cavus (5.9°) compared to the rectus average HAA of 9.8°. These radiographic patterns align with the shape model findings, as both demonstrate CAI morphology trending toward cavus, but in a less severe presentation. Published radiographic studies have similarly reported increased medial arch and hindfoot varus in relation to CAI, further supporting the clinical relevance of our results [8, 15].

Beyond visual and radiographic measurement similarities, the overlap between CAI and cavus morphology likely reflects shared biomechanics. A higher medial arch and hindfoot varus alignment shifts load‐bearing laterally, increasing stress on the lateral ligament complex and reducing the secondary stabilizing potential of the peroneal muscles. These changes in alignment and load distribution may predispose the ankle to recurrent instability events, even in the presence of intact or sufficiently healed ligamentous structures. This is consistent with kinematic and kinetic studies demonstrating impaired ankle eversion strength and delayed peroneal activation in CAI populations [36, 37]. Thus, the osseous alignment identified in this study may not only be a marker of cavus foot type but could also be a contributor to recurrent instability.

The term “subtle cavus” offers a valuable concept for interpreting these findings. Although cavus deformity has been recognized as a risk factor for instability, more recent work suggests that subtle‐cavus features, such as elevated MA and varus hindfoot, may also be relevant [15, 38, 39]. It has also been shown that failure to correct hindfoot varus was the leading cause of unsuccessful ligament reconstruction, contributing to 28% of surgical failures [40]. Together, the SSM results and radiographic measurements emphasize that addressing soft tissue correction alone may not be sufficient. Instead, adjunctive procedures, such as calcaneal and/or medial column osteotomies combined with ligament repair, may be necessary to restore alignment and reduce the risk of recurrent instability [41].

This study has several limitations. The analysis relied on static WBCT imaging, which does not account for dynamic or functional compensatory mechanics. WBCT scans were obtained in CAI patients whose preoperative planning specifically required this type of imaging, potentially introducing a selection bias. Not all patients who have undergone CAI require a WBCT, so the cohort of CAI patients for WBCT represents a subset of patients whose anatomic features may be unique. Future research should include larger populations, the integration of functional assessments, and longitudinal follow‐up of patients with CAI, including those post‐ligament reconstruction, to evaluate outcomes and further define the relationship between cavus morphology, hindfoot alignment, and the risk of CAI.

Recognizing that CAI is similar to cavus foot type reinforces CAI as a condition with both ligamentous and structural alignment components. Recognizing subtle cavus deformities is important when optimizing surgical outcomes. In practice, this means that surgical procedures may need to include soft‐tissue stabilization and realignment procedures to achieve long‐lasting ankle stability.

## Author Contributions

**E. Renae Lapins:** study design, segmentation, statistical shape modeling, data analysis, statistical analysis, manuscript writing. **Alayna Fendler:** segmentation, statistical shape modeling, manuscript editing. **Scott LaTulip:** image data collection, manuscript editing, clinical insight. **Devon C. Nixon:** image data collection, manuscript editing, clinical insight. **Amy L. Lenz:** study design, grant applications, manuscript writing.
